# Loss of Fatty Acid Oxidation by Neural Stem and Progenitor Cells Increases Proliferation but Does Not Improve Long-Term Neurogenesis After Mild Traumatic Brain Injury

**DOI:** 10.1080/17590914.2025.2610198

**Published:** 2026-01-18

**Authors:** Javier Allende Labastida, Regina F. Fernandez, Tiffany Chu, Noelle Puleo, Maria Shishikura, Michael J. Wolfgang, Joseph Scafidi, Susanna Scafidi

**Affiliations:** a Department of Anesthesiology and Critical Care Medicine, Johns Hopkins University School of Medicine, Baltimore, MD, USA; b The Michael V. Johnston Center for Developmental Neuroscience, Kennedy Krieger Institute, Baltimore, MD, USA; c Department of Neurology, Johns Hopkins University School of Medicine, Baltimore, MD, USA; d Department of Physiology, Pharmacology & Therapeutics, Johns Hopkins University School of Medicine, Baltimore, MD, USA; e Department of Pediatrics, Johns Hopkins University School of Medicine, Baltimore, USA

**Keywords:** Fatty acid oxidation, hippocampus, mild traumatic brain injury, neural stem and progenitor cells, proliferation

## Abstract

Neurogenesis in the dentate gyrus of the hippocampus is a conserved and highly regulated process throughout the lifespan. Hippocampal neural stem and progenitor cells (NSPCs) can either transition into an activated proliferative state or remain quiescent. Accumulating data suggests that mitochondrial fatty acid β-oxidation is important in maintaining NSPCs quiescence under normal physiological conditions; however, the contribution of this pathway in NSPCs following brain injury remains unknown. While severe traumatic brain injury (TBI) is characterized by increased NSPCs proliferation in the hippocampus, the extent of this proliferative response after mild TBI, the most prevalent form of TBI, has not been fully delineated. Using closed head injury as a model of mild TBI and a brain-specific knockout mouse of carnitine palmitoyltransferase 2 (CPT2; an obligate gene in mitochondrial fatty acid β-oxidation), we investigated the role of fatty acid oxidation in hippocampal NSPCs proliferation in naïve and injured male and female mice. Our results show that loss of CPT2 in the brain does not affect hippocampal proliferation in naïve mice. Furthermore, mild TBI upregulates proliferation at day 3 post-injury, and is further increased only in male CPT2-deficient mice. Despite the post-injury increase in hippocampal NSPCs proliferation in CPT2^B−/−^ mice, long-term neurogenesis remained unchanged. Together, these data provides a new insight into the metabolic regulation of NSPCs neurogenesis in the hippocampus following mild traumatic brain injury.

## Introduction

TBI is a leading cause of cognitive dysfunction and lifelong morbidity, with 70-87% of the cases classified as mild TBI (mTBI) (Barlow et al., 2010; Cassidy et al., 2004; Dewan et al., 2019; DeWitt et al., 2013; Kane et al., 2011; Laker, 2011; National Center for Injury Prevention & Control, 2015). While moderate and severe TBI typically involve structural brain damage, mTBI is defined by transient neurological symptoms and lack of overt structural abnormalities (National Center for Injury Prevention & Control, 2015). Despite this, mTBI can lead to long-term neurological sequelae, particularly affecting hippocampal tasks such as attention, memory, and learning (Eakin & Miller, 2012; National Center for Injury Prevention & Control, 2003; Rabinowitz & Levin, 2014; Smith et al., 2015; Thornhill et al., 2000). Although the hippocampus is not directly injured by the impact, both clinical and preclinical studies have extensively documented acute hippocampal cell death and long-term volume loss (Cole et al., 2018; Harris et al., 2019; Hicks et al., 1993; Maxwell et al., 2003; Zhou et al., 2012). Over the past decade, accumulating evidence has shown that, similar to other injury paradigms, moderate-to-severe TBI induces proliferation of neural stem and progenitor cells (NSPCs) as an innate repair mechanism (Chirumamilla et al., 2002; Wang et al., 2016; Zheng et al., 2013).

In the adult brain, NSPCs are restricted to specific regions, one of which is the sub-granular zone (SGZ) of the hippocampal dentate gyrus. To date, most studies examining NPSCs response after TBI have been conducted in models of moderate-to-severe TBI and almost exclusively in males (Bielefeld et al., 2024; Chirumamilla et al., 2002; Dash et al., 2001; Gao & Chen, 2013; Rola et al., 2006; Mocciaro et al. 2020). Results of these studies continue to be inconclusive, with some reporting that newly differentiated cells primarily become astrocytes while others suggest that TBI promotes neurogenesis (Bielefeld et al., 2024; Pous et al., 2020). However, the extent and regulation of NSPCs proliferation after mTBI are less well characterized and have been studied only in males (Clark et al., 2020). While these questions remain, it is well established that the transition of stem cells from quiescence to proliferation in many tissues is characterized by a metabolic shift (Shapira and Christofk 2020). In the brain, several studies have shown that NSPCs are dependent on mitochondrial fatty acid oxidation during the quiescent state (Knobloch et al., 2017; Wani et al., 2022).

Brain fatty acid oxidation is a multi-step process that begins with the mitochondrial transport of fatty acids for subsequent β-oxidation within the mitochondrial matrix. This mitochondrial transport is mediated by carnitine palmitoyltransferases (CPTs): CPT1a located on the outer mitochondrial membrane, and CPT2, located in the inner membrane. CPT1a is the rate-setting enzyme that transfers long-chain acyl groups to carnitine to form acylcarnitines, which can traverse the mitochondrial membrane. Once in the mitochondrial matrix, CPT2 converts acylcarnitines to acyl-CoAs, which can be further metabolized by long- and medium-chain dehydrogenases (ACADL and ACADM) and undergo β-oxidation. While *Cpt1* has three known isoforms, only one *Cpt2* exists (Bonnefont et al., 2004). To date, several studies have examined the role of CPT1a in neurogenesis, showing that indeed quiescent NSPCs express the CPT machinery at higher levels than activated NSPCs (Knobloch et al., 2017; Stoll et al., 2015; Xie et al., 2016). In contrast, less is known regarding the role of CPT2 (an obligate gene in fatty acid oxidation) on NSPCs in the adult brain hippocampus. White et al. used pan-brain specific CPT2 knockout mice and showed that disruption of brain fatty acid oxidation elicits robust changes in brain metabolism (White et al., 2020). More recently, Wani et al. reported that genetic ablation of the mitochondrial protease YME1L significantly altered the mitochondrial proteome, resulting in downregulation of the fatty acid oxidation machinery, and negatively affecting NSPCs self-renewal by promoting early differentiation (Wani et al., 2022). These findings underscore the importance of mitochondrial metabolism in regulating NSPCs activity. However, despite evidence that fatty acid oxidation is critical for regulating NSPCs activity, the effects of TBI on this metabolic pathway have not been examined.

Given the role of fatty acid oxidation in maintaining NSPCs quiescence, and the central role of CPT machinery in this process, we hypothesize that the loss of mitochondrial fatty acid oxidation via genetic deletion of *Cpt2* in NSPCs results in increased proliferation in the adult brain and this is further augmented by mild traumatic brain injury. Our results show that loss of CPT2 did not affect proliferation of NSPCs in the naïve adult mouse hippocampus. Mild TBI induced proliferation of hippocampal NSPCs, and loss of CPT2 heightened this proliferative response. However, long-term, there was no increased survival of these newly generated cells.

## Methods

### Animals

All procedures were performed in accordance with the NIH’s Guide for the Care and Use of Laboratory Animals and under the approval of the Johns Hopkins University School of Medicine’s Animal Care and Use Committee. All mice were housed in ventilated racks with a 12-hour light/dark cycle in a temperature and humidity-controlled room, food and water were provided *ad libitum*, with standard chow diet (2018SX; Teklad Global Diets).

The following mouse strains were obtained from The Jackson Laboratory: Nestin-Cre mice (B6.Cg(SJL)-TgN(Nes-Cre)1Kln) (RRID:IMSR_JAX:003771) and B6.129X1-*Gt(ROSA)26Sortm1(EYFP)Cos/J* (RRID:IMSR_JAX:006148). Nestin-Cre transgenic mice were bred with Cpt2^flox/flox^ (Cpt2^f/f^) to generate experimental mice with brain specific loss of fatty acid oxidation (CPT2^B−/−^) as previously described (White et al., 2020). The Cpt2^f/f^ littermates were used as controls. The Nestin-Cre/Cpt2^f/f^ mice were also crossed with ROSA26-YFP expressing reporter mice to create Nestin-Cre/Cpt2^f/f^/Rosa^f/f^ (R26YFP.CPT2^B−/−^). Nestin-Cre/Rosa^f/f^ were used as controls. Both male and female mice were used in these studies.

### TBI – Closed Head Injury Model (CHI)

The CHI procedure followed protocols previously described (Creed et al., 2011). Both male and female mice age 9–10-weeks-old were used in this study. Mice were administered isoflurane anesthesia with 30% oxygen (4% isoflurane for induction and 2% maintenance) and CHI or sham procedures were performed. Mice were placed in a stereotactic device and positioned beneath the pneumatic powered cortical impact device (Pittsburgh Precision Instruments, Pittsburgh, PA, USA). The scalp was prepped with povidone iodine and a 1.0 cm midline rostral-to-caudal incision was made to expose the skull. Mice received CHI using a 5 mm diameter flat metal impactor tip, zeroed at the sagittal suture midway between the bregma and the lambda with a velocity of 4.5 m/s ± 0.3, depth of 1.5 mm, and dwell time of 100msec. Following impact, the incision was closed with a 4-0 proline suture. Sham-injured mice received the same incision and suture but no impact. Immediately after the impact, apnea and righting reflex times were recorded.

### BrdU Injection

To examine the extent of proliferation in control and CPT2^B−/−^ mice, naive mice were injected with bromodeoxyuridine (BrdU) (50 mg/kg) × 3 times two hours apart (q2hrs × 3). CHI mice were injected at 24, 22, and 20 hours before the surgery, and subsequently perfused at 1, 3, and 7 days post-injury. A separate cohort of mice received CHI or sham injury, followed by three injections of BrdU (50 mg/kg, administered two hours apart) at 1, 3, and 7 days post-injury, and were perfused to assess short-term proliferation. Long-term proliferation and survival were assessed at 28 days after injury.

### RNA Analysis

Total RNA was isolated from microdissected hippocampi using Trizol (Life Technologies, Grand Island, NY), purified using the RNeasy Plus Mini Kit (Qiagen; 74134), and quantified by NanoDrop One spectrophotometer (Thermo Fisher Scientific). RNA was converted to cDNA using the High-Capacity cDNA Reverse Transcriptase kit (Applied Biosystems; 4368814). Real-time PCR (RT-PCR) was performed using SsoAdvanced Universal SYBR Green Master Mix (Bio-Rad;1725274) with primers specific for the genes of interest (Supplementary Table 1). RT-PCRs were carried out in a CFX Opus 96 thermocycler (Bio-Rad). All data were normalized to the average of housekeeping threshold cycle (*C_T_*) values from *18S*, *Ppia*, and *Rpl22*. Normalized data were expressed as 2^−ΔCT^ and represented as fold differences.

### Immunohistochemistry

Mice were intracardially perfused with PBS and 4% paraformaldehyde (PFA), brains were fixed in 4% PFA for 24 hrs and subsequently cryoprotected with 30% sucrose in PBS. Brains were sectioned coronally on a sliding microtome at 30 µm. For histological analyses, three sections (spaced 300 μm apart) that contained the hippocampus were selected and a free-floating staining protocol was followed. Briefly, the sections were rehydrated and washed 3 times, 10 mins each, in 1 × PBS, followed by a 15-minute incubation in 3% H_2_O_2_ (Millipore Sigma, H1009). After another 3 washes in 1 × PBS, we performed antigen retrieval for 25 mins in 2 N HCl and then 15 mins in 1.5 mM boric acid (pH = 8.5). After 3 more washes in 1× PBS, the sections were blocked using 20% donkey serum in carrier solution (1% BSA, 0.3% Triton X-100 in 1 × PBS) for 1 hr at room temperature (RT). Brain sections were then incubated with primary antibodies in carrier solution overnight at 4 °C. The next day, sections were washed 6 times with 1 × PBS and then incubated for 2 hrs with the secondary antibodies in carrier solution. After another 6 washes, the tissues were placed in DAPI (1:1000) (Invitrogen, D1306) for 10 mins, rinsed in 1× PBS, mounted onto a slide, and coverslipped using Prolong Gold Antifade (Invitrogen, P36930). The primary and secondary antibodies used are listed in Supplementary Table 2. Images of brain sections were acquired with a Leica SP8 laser scanning confocal microscope and a DMI8 epifluorescence microscope with Leica-DFC9000GT-VSC13457 camera. Confocal imaging was acquired using the tilescan function, 2 × 3 or 4 fields of view were imaged at 20× (HC PL APO CS2 20×/0.75 DRY) and Z-stacks were acquired for a thickness of 20 μm with 2 μm step size. Z-stacks of the 2–3 fields of view where then merged using Leica LAS X software (v3.7.6.25997). For proof of co-localization, images were acquired with an oil immersion objective at 100× (using a 63× objective, HC PL APO CS2 63×/1.40 OIL, with 1.6 zoom factor). 25 μm thick z-stacks were taken with 1 μm steps and orthogonal sectioning of the images using Leica LAS X software. Epifluorescence images were acquired using navigator function with a 20× objective, (HC PL APO CS2 20×/0.80 DRY). Merged hippocampi were imaged at 20× requiring 4–8 fields of view, post-processing was done using the Leica LAS X software (v3.7.6.25997). All quantification was done by a blinded investigator using Imaris v9.5 (Bitplane, Zurich, Switzerland).

### Behavioral Testing

Prior to any behavioral testing, mice were accustomed to the housing facility for 7 days. Following habituation, a health and reflex assessment was performed. Mice 10–11-weeks-old were habituated to the experimenter by handling for 5 days, on the last two days of handling, mice were transported and handled in the behavior room to habituate them to the transport. All behavior tests were performed between 9:00 am and 4:00 pm during the light portion of the light/dark cycle. Mice were allowed to habituate to the testing room for at least 30 min before any testing, returned to the animal colony before the beginning of the dark cycle, and any home cage changes were done at least 24 hours before any behavior test. Behavioral tests were carried out in the following order: Y-maze spontaneous alternation, open field, novel location recognition (NLR), novel object recognition (NOR), SHIRPA, rotarod, and Y-maze spatial recognition. Behavioral scoring was unbiased, blinded and automated with the use of ANY-maze video tracking software (v6.3, Stoelting Co., Wood Dale, Il). Rotarod was performed with the Rotamex apparatus (Columbus instruments, Columbus, OH) and hand scored by a blinded observer. Experimenter was blinded to genotype but not sex.

- Y-maze spontaneous alternation was used to assess spatial working memory. Mice were placed in the center of a Y-maze and allowed 5 minutes to freely explore all 3 open arms. The number and order of entries into the arms of the maze were counted, considering correct alternations only those that included all three arms without repetition, regardless of the sequence. Percent of correct alternation was calculated by diving the number of correct alternations by the number of potential alternations [(number of entries –2) × 100] (Ijomone et al., 2015).
- Open field test. Each mouse was exposed to the open field arena to measure locomotion activity and anxiety like behavior. Briefly, mice were placed in the center of the open field (40 × 40 × 40 cm, Stoelting Co, Wood Dale, IL, USA) and allowed to freely explore for 10 min. Digital recording and quantification of the behavior was carried out with Any Maze software. Peripheral area included a 10 cm wide perimeter and the internal remainder area was the center. The following parameters were assessed: total distance traveled, average speed, time and distance in periphery vs center and thigmotaxis index as assessment of anxiety. Arena was cleaned with 50% alcohol (v/v) in between animals to eliminate odor cues. Open field procedure was repeated in the afternoon and twice the following day to reduce the novelty of the arena and increase object exploration in novel location and novel object recognition tests.
- Novel location recognition (NLR) was used to assess hippocampal dependent spatial recognition memory. Briefly, following the second day of open field, mice were subjected to the arena with the addition of two objects. Objects were placed on the far side of the arena 10cm apart from each of two walls in the corners. Mice were then released on the proximal medial corner of the arena and allowed to explore freely for 10 min, after which they were removed from the arena and returned to their home cage. After an intertrial period of 24 hours, mice were reintroduced to the arena, however, one of the objects was displaced (object distal to the release corner), and mice were allowed to explore freely, quantification of exploration times on the objects was recorded, and mice were returned to their home cage. Discrimination index was calculated as exploration of novel location/total exploration of the objects × 100 and reported as percent. Cleaning of the arena and objects was performed as described in the open field. To decrease the novelty generated by the novel location, a second exposure to the objects was done in the afternoon.
- Novel object recognition (NOR) was used to assess recognition memory. Briefly, 24 hours after novel location recognition, mice were reintroduced in the arena. This time the object in the familiar location was changed for a novel object. Mice were allowed to freely explore the objects for 10 min. Quantification and discrimination index were calculated as mentioned above. Arena and objects were cleaned in between animals as described above.
- SHIRPA. A general health assessment and neurological screening test validated for the phenotypic characterization of transgenic mice was performed (Hatcher et al., 2001; Masuya et al., 2005). Briefly, the mice were placed in a cylindrical arena and observed for their physical characteristics (coat color, head, eye, ear, tail and limb morphology, body position, size and weight) and their behavior (spontaneous activity, respiration, tremors) were assessed with an arbitrary scale (4 = good, 3 = fair, 2 = moderate, 1 = low and 0 = none). This assessment was followed by the evaluation of exploratory behavior on a large box (50x30x30cm), including, locomotor activity, transfer arousal, piloerection, startle response, gait and escape. Finishing with provoked activity, including, positional passivity, trunk curls, visual placing and limb grasping, body tone, wire maneuver, and recording the responses to a series of reflex tests, including fear, irritability, aggression and vocalization.
- Rotarod was used to assess motor function, coordination and balance. Naïve Cpt2^f/f^ and CPT2^B−/−^ mice age 12–13-weeks-old were tested using Rotamex apparatus (Columbus instruments, Columbus, OH). Mice were acclimatized to the apparatus by being exposed to a stationary rod for 30 seconds, followed by the rotating rod at a constant speed of 4.0 RPMs for 30 seconds. If the mice accomplished these tasks without falling, they carried out the rotarod protocol after a 30 min intertrial resting period. Each mouse was placed in the rod, and once the mouse was in the rod, the trial was initiated at 4.0 RPM and rotated at this speed for 5 seconds, after which the rod underwent a constant acceleration of 0.12 RPM per second up to 295 seconds, reaching a top speed of 39.4 RPM at 5 minutes. Latency to fall was recorded. In between trials, the rotarod was cleaned using damp paper towel with 70% alcohol, and allowed to dry.
- Y-maze spatial recognition was used to test recognition memory in mice. This test is composed of two stages, in stage 1 mice were placed in the Y-maze in which one arm was blocked from entry and mice were allowed to explore freely for 5 min. After an inter-stage period of at least 30 min, mice were re-introduced into the Y-maze but the blockade was removed. Mice were allowed to freely explore the maze and time and distance covered in each arm was quantified.

### Statistical Analysis

All investigators responsible for data collections and analysis were blinded to genotype and injury. Data shown as the mean ± SEM for each group unless otherwise stated. Data was analyzed using GraphPad Prism10 software (Boston, MA). Sample size for each experiment is detailed in each figure legend. Two-tailed Student’s *t* test, two-way ANOVA (injury, genotype), or 2-way mixed-effect model (REML) ANOVA with Bonferroni *post-hoc* analysis was used to determine statistically significant differences between the groups. A *p* < 0.05 was considered statistically significant.

### Artwork

Experimental design figures were created with BioRender.com full license plan.

## Results

### Loss of Mitochondrial Fatty Acid Oxidation via Deletion of Cpt2 Does Not Increase Proliferation in Neural Progenitor Cells

**Figure 1.**
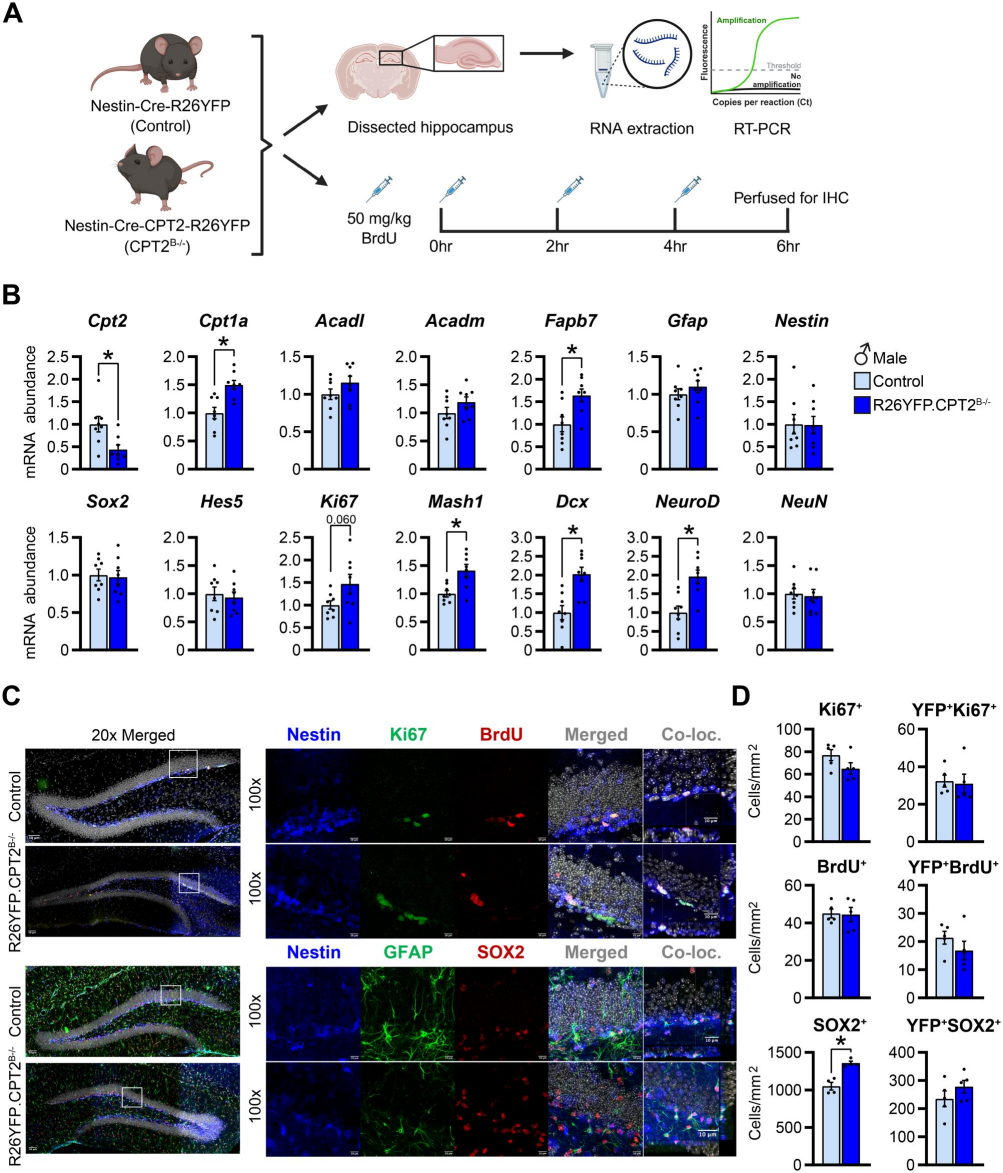
Loss of fatty acid oxidation in neural progenitor cells does not increase proliferation in the adult dentate gyrus. (A) Experimental design to determine the effect of the loss of CPT2 in the brain under basal conditions: 9–10 week-old-naïve Nestin-Cre-R26YFP (control) and Nestin-Cre-CPT2-R26YFP (R26YFP.CPT2^B−/−^) male mice were used. Gene expression from dissected hippocampus was measured by RT-PCR. For histological analysis, control and R26YFP.CPT2^B−/−^ mice were injected with BrdU (50 mg/kg, q2hrs ×3) and then perfused. (B) mRNA abundance of genes associated with fatty acid oxidation (*Cpt2*, *Cpt1a*, *Acadl*, *Acadm*, *and Fabp7*) and neurogenesis (*Gfap*, *Nestin*, *SOX2*, *Hes5*, *Ki67*, *Mash1*, *Dcx*, *NeuroD*, and *NeuN*) in hippocampus of male mice expressed as fold change from control; *n* = 8/group. (C) Representative images of immunostaining in the dentate gyrus of control and R26YFP.CPT2^B−/−^ male mice. Top panel: Nestin-Rosa-YFP (blue), Ki67 (green), and BrdU (red). Bottom panel: Nestin-Rosa-YFP (blue), GFAP (green), and SOX2 (red). Scale bars, 50µm (20x magnification), 10µm (100× magnification). (D) Quantification of Ki67^+^, YFP^+^Ki67^+^, BrdU^+^, YFP^+^Brdu^+^, SOX2^+^, and YFP^+^SOX2^+^ cells; *n* = 4–5/group. Data are presented as mean ± SEM. Statistical analysis for (B,D) by unpaired 2-tailed Student’s t test; *p < 0.05 (For (B), *Cpt2 p* = 0.0140, *Cpt1a p* = 0.0018, *Fabp7 p* = 0.0105, *Mash1 p* = 0.0046, *Dcx p* = 0.0018, *NeuroD p* = 0.0015; for (D), SOX2^+^ *p* = 0.0015). *Cpt2*: carnitine palmitoyltransferase 2; *Cpt1a*: carnitine palmitoyltransferase; *Acadl*: acyl-CoA dehydrogenase long chain; *Acadm*: acyl-CoA dehydrogenase medium chain; *Fabp7*: fatty acid binding protein 7; *Gfap*:glial fibrillary acidic protein; *SOX2*: SRY-box transcription factor 2, *Hes5*: hes family bHLH transcription factor 5; *Mash1*: achaete-scute family bHLH transcription factor 1; *Dcx*:doublecortin; *NeuroD*: neurogenic differentiation 1; *NeuN*: neuronal nuclei antigen; Co-loc: co-localization. Panel (A) was created in BioRender. Scafidi, S. (2025) https://BioRender.com/mbc8onb

*Cpt2* is the obligate gene for mitochondrial fatty acid oxidation of long-chain fatty acids. To study the role of CPT2 in NSPCs in the adult brain hippocampus, we generated mice with a brain-specific knockout of CPT2 using the Nestin promoter Cre driver (CPT2^B−/−^) (White et al., 2020). These mice were then crossed with ROSA26-YFP expressing reporter mice (controls) to produce Nestin-Cre-CPT2-R26YFP mice (R26YFP.CPT2^B−/−^) (Figure 1A). We confirmed deletion of CPT2 in the brain of male and female mice by performing gene expression analysis in the hippocampus (Figure 1B and S1A). In addition, we assessed whether loss of CPT2 resulted in transcriptional changes of other genes involved in fatty acid metabolism. *Cpt2* deletion led to an upregulation of *Cpt1a* mRNA in both sexes. No transcriptional changes were observed in the expression of long- and medium-chain acyl-CoA dehydrogenases (*Acadl* and *Acadm*) in the hippocampi of these mice compared to controls (Figure 1B and S1A). Fatty acid binding protein 7 (*Fabp7*), an enzyme that regulates fatty acid uptake and transport and has been shown to be involved in neurogenesis, was upregulated in R26YFP.CPT2^B−/−^ males but not in females compared to controls (Figure 1B and S1A) (Matsumata et al., 2012; Storch & Corsico, 2023).

Next, we evaluated the effect of CPT2 deficiency in the brain on the transcription of genes related to neurogenesis. The expression of glial fibrillary acidic protein (*Gfap),* expressed in quiescent NSPCs in the adult hippocampus and in mature astrocytes was not altered by the loss of CPT2 in the brain (Figure 1B and S1A). Some markers of proliferation and/or differentiation remained unchanged in R26YFP.CPT2^B−/−^ compared to controls (*Nestin*, *SOX2*, SRY-box transcription factor 2; and *Hes5*, hes family bHLH transcription factor 5) while others were upregulated or trending in R26YFP.CPT2^B−/−^ males but not in females (*Ki67*, marker of proliferation Kiel 67; *Mash1,* achaete-scute family bHLH transcription factor 1*; Dcx,* doublecortin*;* and *NeuroD,* neurogenic differentiation 1) (Figure 1B and S1A). Furthermore, the marker of mature neurons, *NeuN*, was not changed (Figure 1B and S1A). Together, these data indicate that under normal conditions, loss of brain CPT2 results in the upregulation of some neurogenic genes in the hippocampus of males only.

To determine the effect of *Cpt2* genetic deletion on NSPCs proliferation, we injected BrdU (50 mg/kg – 2 hrs × 3) to control and R26YFP.CPT2^B−/−^ mice to label actively proliferating cells in the dentate gyrus of the hippocampus (Figure 1A). We performed immunostaining of Ki67, BrdU, GFAP, and SOX2 that belong to the Nestin-Rosa-YFP neural stem cell lineage and their progeny (Figure 1C). Quantification of YFP^+^Ki67^+^ cells revealed no differences in the number of nestin-positive NSPCs undergoing mitosis (YFP^+^Ki67^+^) between control and R26YFP.CPT2^B−/−^ male mice (Figure 1D). In females, the number of YFP^+^Ki67^+^ cells showed a tendency to decrease in R26YFP.CPT2^B−/−^ mice (*p* = 0.055) (Figure S1B). Furthermore, the loss of CPT2 did not affect the number of YFP^+^BrdU^+^ cells, actively dividing cells that are in the S-phase of the cell-cycle during the injection (Figure 1D and S1B). The number of NSPCs positive for Nestin and SOX2 (YFP^+^SOX2^+^) was similar between control and R26YFP.CPT2^B−/−^ male mice and trended towards an increase in female R26YFP.CPT2^B−/−^ mice (*p* = 0.051) (Figure 1D and 1SB). Taken together, these data demonstrate that loss of functional CPT2 in the brain of naïve mice does not significantly alter the number of hippocampal NSPCs in the proliferative state.

### Loss of CPT2 in the Brain Does Not Affect Long-Term Behavioral Outcomes

**Figure 2.**
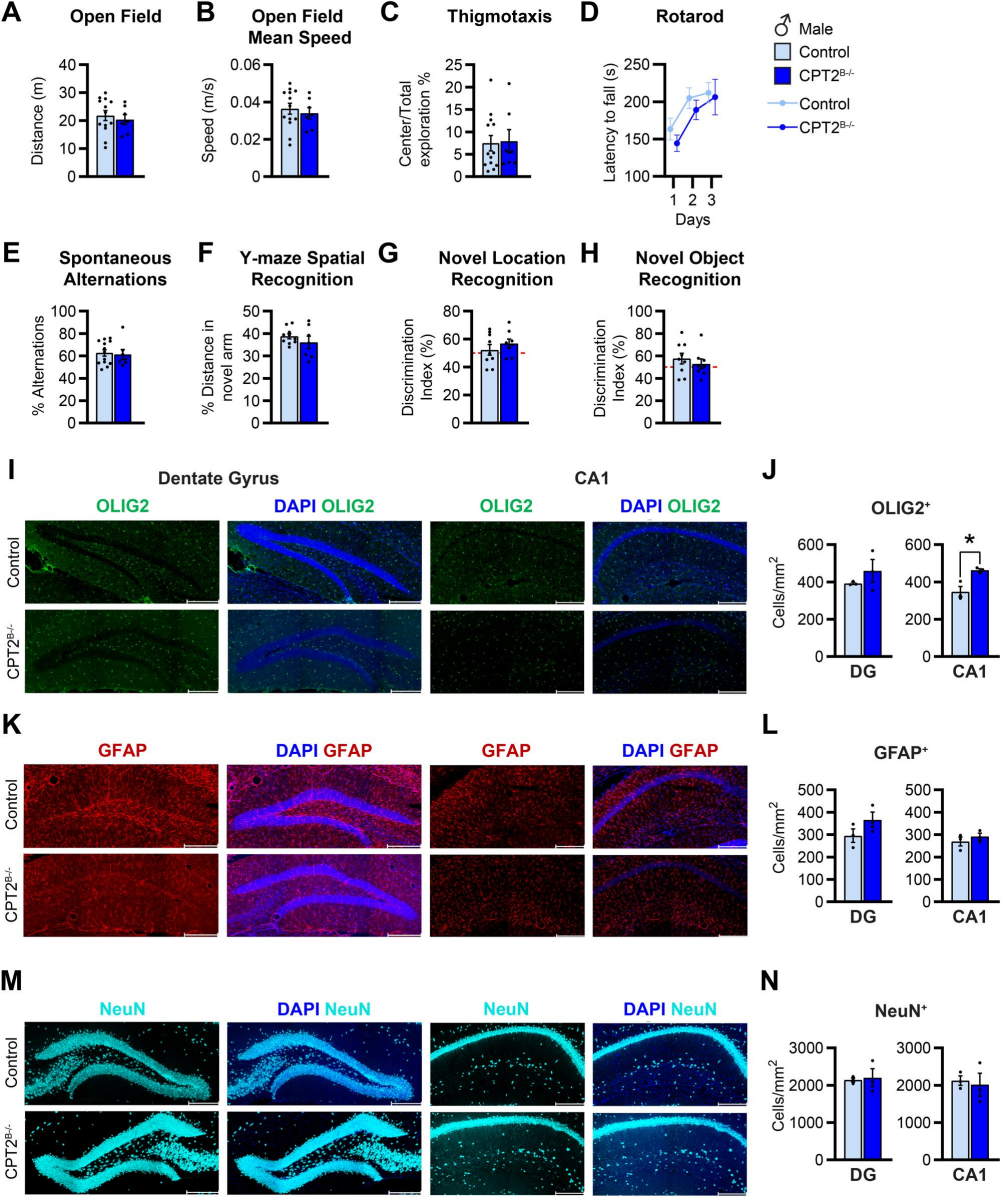
CPT2^B−/−^ mice display a similar behavioral phenotype compared to controls. (A) Total distance traveled, (B) mean speed, and (C) thigmotaxis of control and CPT2^B−/−^ males in an open field; *n* = 7–13/group. (D) Latency to fall from the rotarod for control and CPT2^B−/−^ males; *n* = 7–10/group. (E) Y-maze percent of correct spontaneous alternations of control and CPT2^B−/−^ males; *n* = 7–13/group. (F) Percent distance traveled in the novel arm during the Y-maze spatial recognition test for control and CPT2^B−/−^ males; *n* = 7–10/group. (G) Discrimination index during the novel location recognition test for control and CPT2^B−/−^ males; *n* = 8–9/group. (H) Discrimination index during the novel object recognition test for control and CPT2^B−/−^ males; *n* = 8–9/group. (I,K,M) Representative images of immunostaining in the dentate gyrus and CA1 regions of the hippocampus in control and CPT2^B−/−^ male mice of DAPI (blue) and (I) OLIG2 (green), (K) GFAP (red), and (M) NeuN (cyan). Scale bar 200µm (20× magnification). (J,L,N) Quantification of (J) OLIG2^+^ cells per mm^2^, (L) GFAP^+^ cells per mm^2^, and (N) NeuN^+^ cells per mm^2^; *n* = 3/group (5–6 slices per mouse). Data are presented as mean ± SEM. Statistical analysis for (A-H,J,L, and N) by unpaired 2-tailed Student’s t test; *p < 0.05 (For (J), OLIG2^+^ *p* = 0.0174).

NSPCs play a central role in maintaining brain function during development and adulthood. Thus, altering the metabolism of NSPCs by removing fatty acid oxidation may impact the behavior of CPT2^B−/−^ mice. Here we extended previous work and assessed the effects of loss of CPT2 on behavior in 10–12-weeks-old male and female mice (White et al., 2020). We performed a comprehensive phenotypic characterization of CPT2^B−/−^ mice following the SHIRPA protocol (Supplemental Table 3) (Hatcher et al., 2001; Lalonde et al., 2021; Masuya et al., 2005). Loss of CPT2 in the brain did not affect brain weight in either sex, while body weight was lower in males only compared to controls (Figure S2A–S2C). Our results revealed subtle changes in motor behavior only in CPT2^B−/−^ male mice compared to controls including a decrease in locomotor activity and transfer arousal in the arena, as well as a deficiency in the wire maneuver task (Supplemental Table 3). Moreover, we conducted the open field test to determine if the loss of CPT2 in the brain affected locomotor activity and anxiety-like behavior in mice (Figure 2A–C and S2D–F). The distance and speed were not changed in CPT2^B−/−^ mice compared to controls (Figure 2A,B and S2D,E). Thigmotaxis, an index of anxiogenic behavior measured by the amount of time mice remain close to the walls while avoiding the center of the arena, was increased in CPT2^B−/−^ females, consistent with previously published data (Figure 2C and S2F) (La-Vu et al., 2020; Simon et al., 1994; White et al., 2020). In addition, we assessed coordination and balance by rotarod. CPT2^B−/−^ mice latency to fall from the rotarod was similar to controls (Figure 2D and S2G). These data show that deletion of *Cpt2* in the brain results in minimal alterations in motor function.

NSPCs are present in the dentate gyrus of the hippocampus, an area of the brain essential for memory function. To determine if the loss of CPT2 from NSPCs affects memory, we performed several behavioral paradigms that test different aspects of hippocampal dependent memory. Our results show that CPT2^B−/−^ mice performed similar to controls in the Y-maze spontaneous alternation, the Y-maze spatial recognition, and the novel location recognition tasks, which test for spatial and recognition memory (Figure 2E–G and S2H,J). In the novel object recognition test, female but not male CPT2^B−/−^ mice spent less time exploring the novel object, indicative of impaired non-spatial recognition memory (Figure 2H and S2K). The latter finding in females led us to question whether the changes in non-spatial recognition memory could be due to changes in mature cells in the hippocampus of CPT2^B−/−^ mice.

Since NSPCs are multipotent cells that can differentiate into various cell types including oligodendrocytes, astrocytes, and neurons, we sought to determine whether the loss of fatty acid oxidation affected the number of cells in the hippocampus, specifically in the dentate gyrus and the CA1 region. Histological analysis revealed no differences between groups in the number of oligodendrocytes (OLIG2^+^) in the dentate gyrus and an increase in the CA1 region only in male CPT2^B−/−^ mice compared to controls (Figure 2I,J and S2L,M). Loss of CPT2 did not affect the number of astrocytes (GFAP^+^) in either of the two regions (Figure 2K,L and S2N,O). The number of neurons (NeuN) was similar in the dentate gyrus and CA1 region in CPT2^B−/−^ males compared to controls (Figure 2M,N). Female CPT2^B−/−^ mice exhibit a decrease in neural cells in the dentate gyrus but not in the CA1 region (Figure S2P,Q). Altogether, our results suggest that under basal conditions, loss of fatty acid oxidation in the brain does not have significant long-term effects on hippocampal-dependent memory and on the number of NSPCs-derived mature cells in the hippocampus.

### Loss of Mitochondrial Fatty Oxidation Further Increases NSPCs Proliferation after mTBI

Mild traumatic brain injury is the most prevalent type of brain trauma and can have a severe impact on cognitive functions such as learning and memory (Cassidy et al., 2004; National Center for Injury Prevention & Control, 2003; Rabinowitz & Levin, 2014). While numerous studies have shown that moderate-to-severe TBI increases proliferation of NSPCs in the dentate gyrus of the hippocampus in the adult brain, only a handful of studies have examined the extent of proliferation following mTBI (Chirumamilla et al., 2002; Wang et al., 2016; Zheng et al., 2013). Recent works have demonstrated that the fatty acid oxidation pathway is a prominent feature of NSPCs in quiescence and is upregulated in the brain with age and following moderate-to-severe TBI (Fernandez et al., 2025; Morant-Ferrando et al., 2023). Hence, we asked the question whether loss of fatty acid oxidation affects proliferation and survival of newly generated cells post-mTBI.

**Figure 3.**
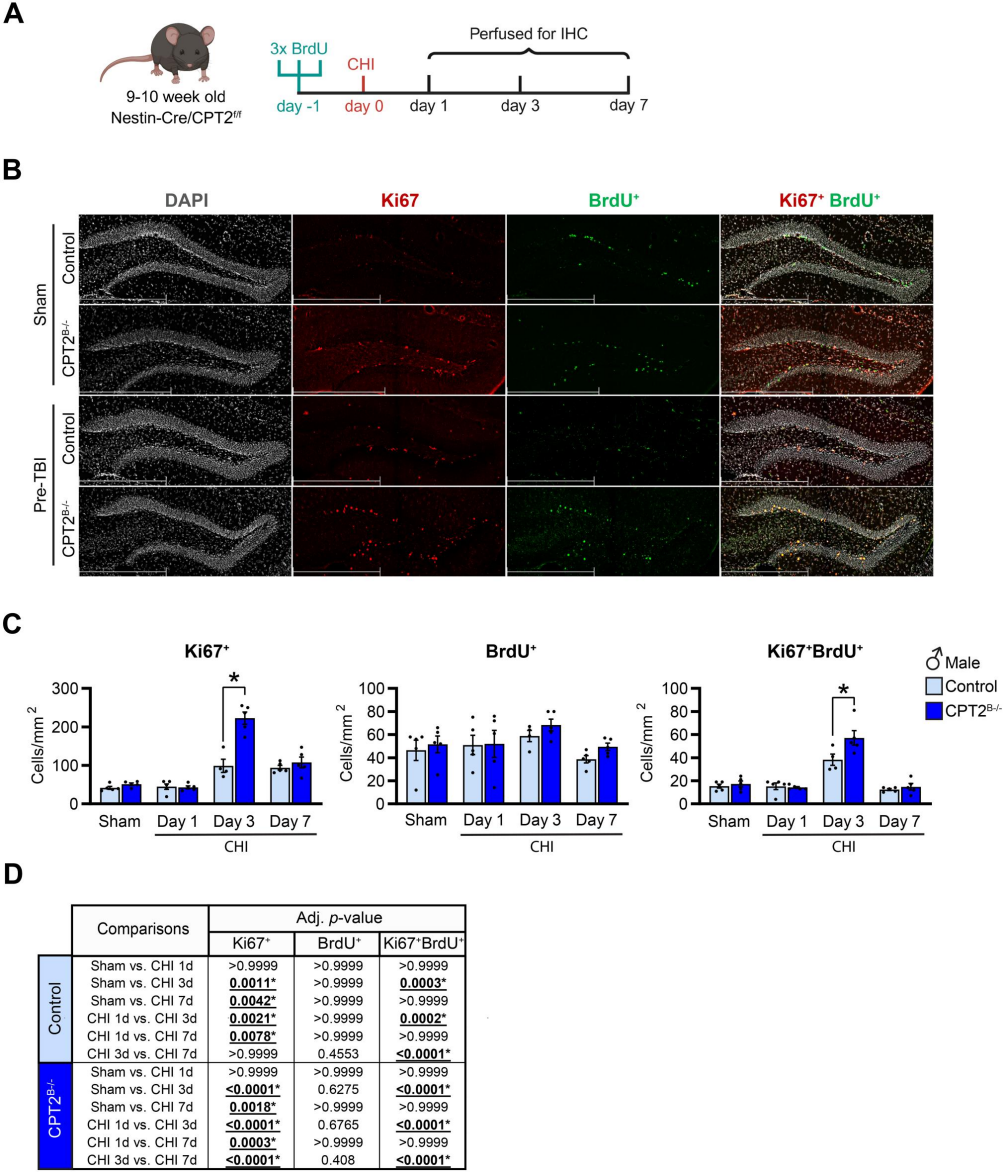
Loss of CPT2 in the brain does not affect the short-term survival and proliferation of pre-labeled NSPCs after CHI. (A) Experimental design to determine the short-term survival of NSPCs after CHI: 9–10-week-old CPT2^f/f^ (control) and Nestin-Cre-CPT2^f/f^ (CPT2^B−/−^) male mice were injected with BrdU (50mg/kg 2hrs ×3) 24 hours prior to receiving CHI or sham surgery. Mice were perfused for histological analysis on day 1, 3, and 7 post-CHI. (B) Representative images of immunostaining in the dentate gyrus (3dpi) of control and CPT2^B−/−^ male mice of DAPI (grey), Ki67 (red), and BrdU (green). Scale bar 200µm (20x magnification). (C) Quantification of Ki67^+^, BrdU^+^, and Ki67^+^BrdU^+^ cells; *n* = 4–5/group/genotype. Data are presented as mean ± SEM. Statistical analysis by 2-way mixed-effect model (REML) ANOVA with Bonferroni *post-hoc* analysis; *p < 0.05 (For (C), Ki67^+^ *p* = <0.0001, Ki67^+^BrdU^+^ *p* = 0.0002). (D) Table containing the adjusted *p*-values from the multiple comparisons performed to determine differences from injury within groups on (C). Panel (A) was created in BioRender. Scafidi, S. (2025) https://BioRender.com/gy0gxbw

First, we examined whether loss of brain CPT2 affected the survival of pre-injury NSPCs after mTBI. We injected mice with BrdU (marker of S-phase of cell proliferation at the time of injection) 24 hours prior to receiving CHI or sham surgery and assessed survival of newly generated cells at 1, 3, and 7 days after the injury (Figure 3A). No significant changes in the number of BrdU^+^ cells were observed in the dentate gyrus of CPT2^B−/−^ mice compared to controls at any time point (Figure 3B–D and S3A,B). Interestingly, there was an increase in Ki67^+^ (marker of G-phases of proliferating cells) at day 3 and 7 post-injury in control male mice compared to sham post-CHI (Figure 3C,D). At day 3 post-CHI, in male CPT2 deficient mice, the number of Ki67^+^ cells and Ki67^+^BrdU^+^ cells were even further augmented compared to injured control and sham mice (Figure 3B,C). In control and CPT2^B−/−^ injured female mice, Ki67^+^ cells were increased on day 7 post-CHI compared to sham (Figure 3SA,B). Together, these data suggest that mTBI does not diminish the survival of pre-injury proliferating cells in both males and females.

**Figure 4.**
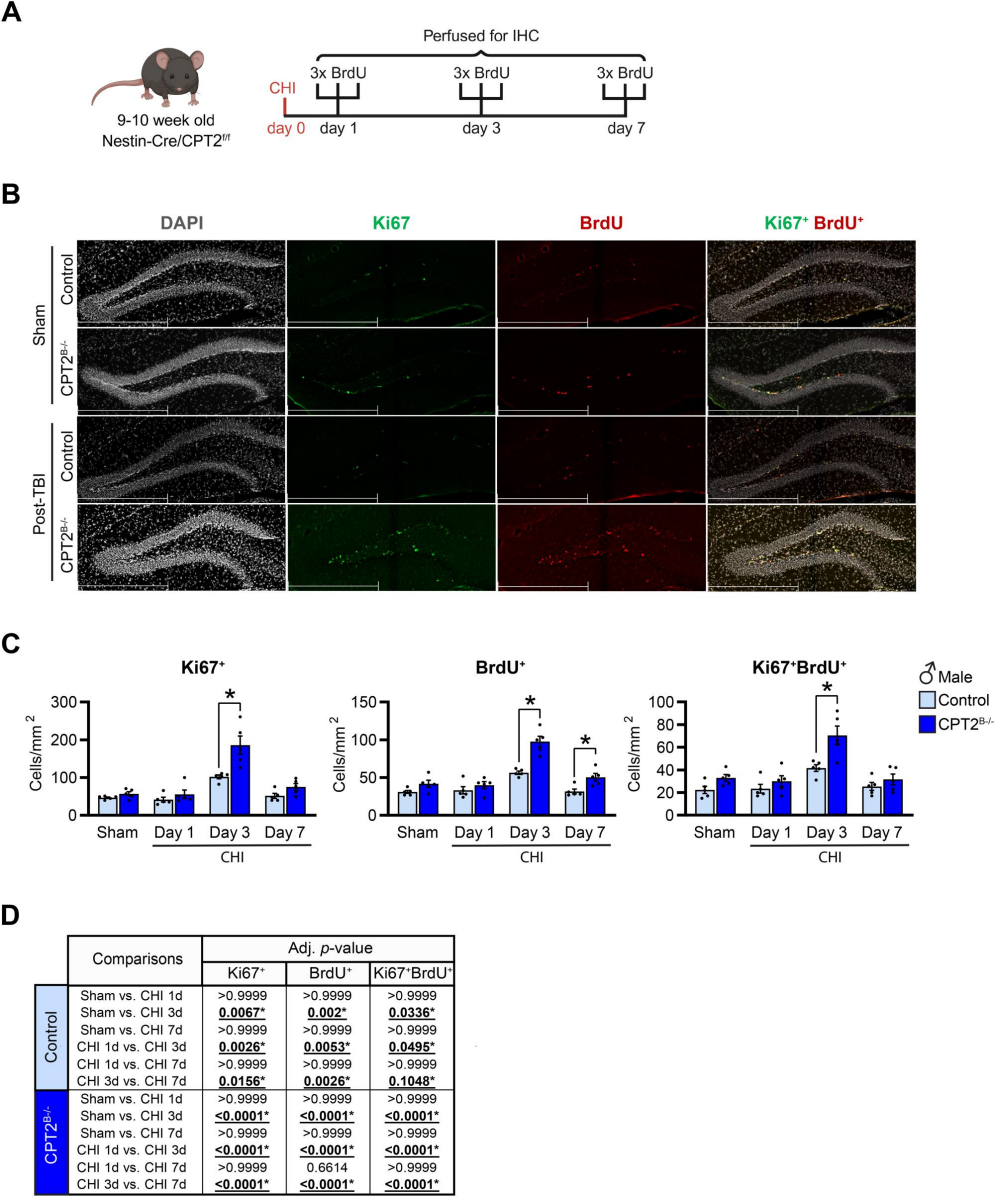
*Cpt2* deletion from the brain further enhance the proliferative response of cells after CHI. (A) Experimental design to determine the proliferative response of cells to CHI: 9-10-week-old CPT2^f/f^ (control) and Nestin-Cre-CPT2^f/f^ (Cpt2^B−/−^) male mice received CHI or sham surgery. Mice were injected with BrdU (50mg/kg q2hrs ×3) and perfused on day 1, 3, and 7 post-CHI. (B) Representative images of immunostaining in the dentate gyrus (3dpi) of control and CPT2^B−/−^ male mice of DAPI (grey), Ki67 (green), and BrdU (red). Scale bar 200µm (20× magnification). (C) Quantification of Ki67^+^, BrdU^+^, and Ki67^+^BrdU^+^ cells; *n* = 4–5/group/genotype. Data are presented as mean ± SEM. Statistical analysis by 2-way ANOVA with Bonferroni *post-hoc* analysis; *p < 0.05. (For (C), Ki67^+^ p ≤ 0.0001, BrdU^+^ Day 3 *p* ≤ 0.0001 and Day 7 *p* = 0.0366, Ki67^+^BrdU^+^ *p* = 0.0031). (D) Table containing the adjusted p-values from the multiple comparisons performed to determine differences from injury within groups on (C). Panel (A) was created in BioRender. Scafidi, S. (2025) https://BioRender.com/6abgiwh

Since we observed increased number of Ki67^+^ cells, we further explored whether CPT2 deficiency in the brain resulted in an augmented increase in cell proliferation after CHI (Figure 3C). Control and CPT2^B−/−^ mice were injected with BrdU at either 1, 3, or 7 days after CHI and brains were collected the same day of the injection for histological analysis (Figure 4A). The number of Ki67^+^, BrdU^+^, and Ki67^+^BrdU^+^ cells was increased in the dentate gyrus of the hippocampus of control injured male mice on day 3 post-injury compared to the other time points and to sham (Figure 4B–D). With the loss of CPT2, the number of Ki67^+^ cells in the dentate gyrus increased 3 days after the injury, while the number of BrdU^+^ cells were increased at day 3 and day 7 post-injury in male mice compared to injured controls (Figure 4B,C). Thus, co-labeling with Ki67 and BrdU was increased at day 3 post-CHI in male CPT2^B−/−^ mice compared to injured controls (Figure 4B,C). Female CPT2^B−/−^ mice did not show differences in Ki67^+^, BrdU^+^, and Ki67^+^BrdU^+^ cells at any time point post-injury compared to injured controls (Figure S3C,D). Together, our results demonstrate that in males, CHI upregulates NSPCs proliferation in the dentate gyrus 3 days post-injury, and this effect is further amplified by the loss of CPT2 in the brain, whereas in females this proliferative response is not observed.

**Figure 5.**
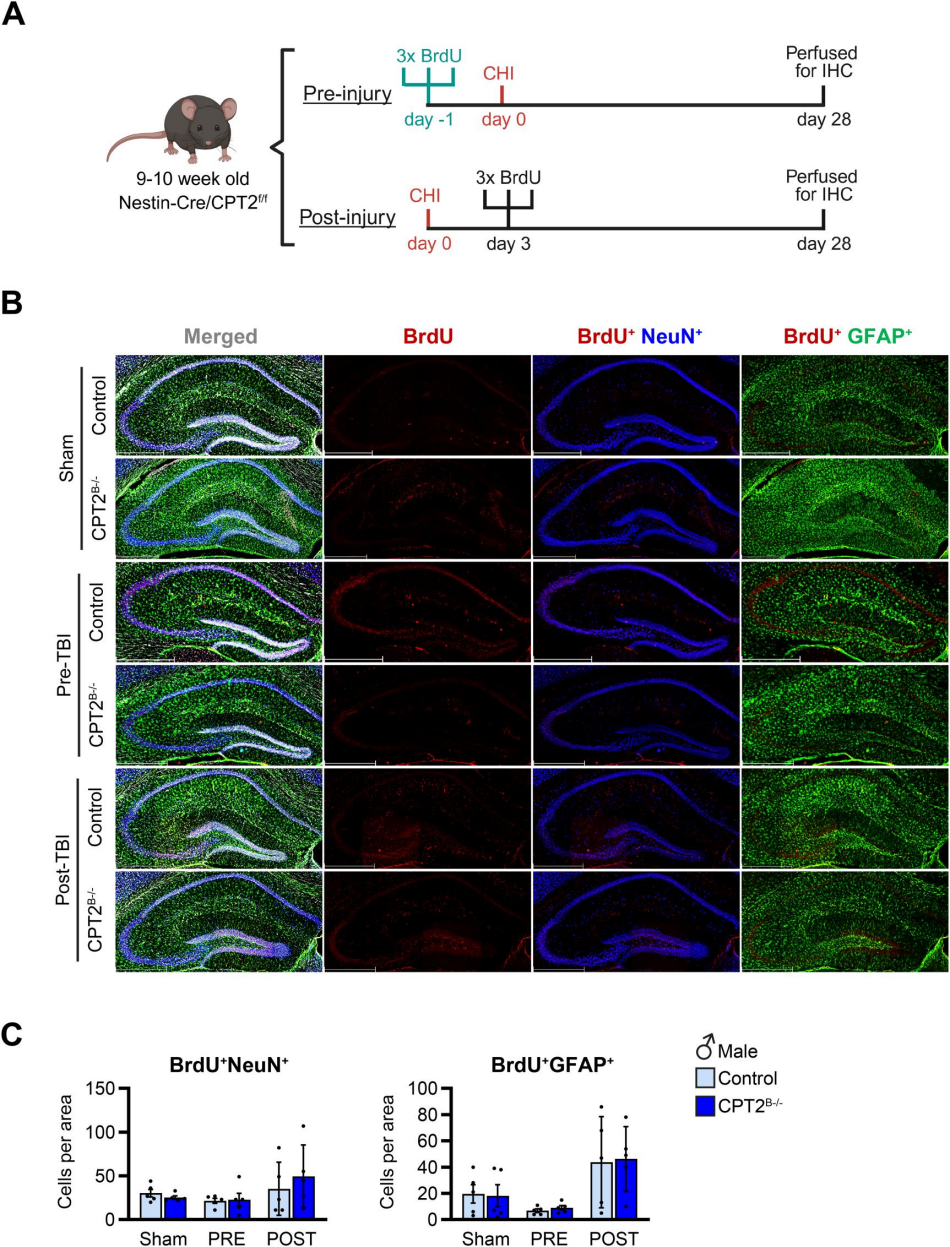
Loss of fatty acid oxidation in the brain does not affect survival 28 days after CHI. (A) Experimental design to determine the fate of proliferative cells after CHI: 9-10-week-old CPT2^f/f^ (control) and Nestin-Cre-CPT2^f/f^ (CPT2^B−/−^) male mice were injected with BrdU (50mg/kg q2hrs ×3) 24 hours prior (PRE) to receiving CHI or sham surgery or injected on day 3 post-injury (POST) and perfused for histological analysis on day 28 post-injury. (B) Representative images of immunostaining in the hippocampus of sham and control and Cpt2^B−/−^ male mice following CHI of BrdU (red), NeuN (blue), and GFAP (green). Scale bar, 200µm (20× magnification). (C) Quantification of BrdU^+^NeuN^+^ and BrdU^+^GFAP^+^ cells in the hippocampus following CHI at 28 days post injury; *n* = 5/group. Data are presented as mean ± SEM. Statistical analysis by 2-way ANOVA with Bonferroni *post-hoc* analysis; *p < 0.05. Panel (A) was created in BioRender. Scafidi, S. (2025) https://BioRender.com/53k0wtl

To determine the long-term fate of these post-injury newly generated cells we performed histological analysis at 28 days post-CHI. We injected control and CPT2^B−/−^ mice with BrdU one day prior to CHI (PRE), to assess the fate of proliferative cells that survive to the CHI, or 3-days post-injury (POST), the time point after CHI when most of the proliferation occurred (Figure 5A). In both male and females, the number of BrdU^+^NeuN^+^ (neurons) and BrdU^+^GFAP^+^ (astrocytes) derived from cells proliferating before CHI did not differ from shams (Figure 5B,C and S3E). Although we saw an increase in proliferation post-CHI, at 28 days post-injury (long-term), the number of BrdU^+^NeuN^+^ and BrdU^+^GFAP^+^ cells did not differ from sham (Figure 5B,C and S3E). Furthermore, while short-term loss of CPT2 resulted in further increase in proliferation, at 28 days, the number of BrdU^+^NeuN^+^ and BrdU^+^GFAP^+^ cells was similar to control injured and sham mice. Altogether, our data shows that despite the augmented proliferative response of cells observed in CPT2^B−/−^ following CHI, the long-term survival of these cells was not improved.

## Discussion

In this study we show that loss of CPT2 – an obligatory enzyme for mitochondrial long chain fatty acid β-oxidation – did not affect proliferation of neural stem and progenitor cells in the naïve adult mouse hippocampus. Mild TBI induced proliferation of hippocampal NSPCs, and loss of CPT2 further augmented this proliferation. However, long-term, this increased NSPCs proliferation did not result in higher survival of the cells generated post-injury.

Recent advances in neurobiology have shown that the brain is capable of self-renewal by activating proliferation and subsequent differentiation of NSPCs. Adult neurogenesis, which occurs primarily in the dentate gyrus of the hippocampus, is a regulated process. Numerous studies have reported an array of markers (proteins) specific for each stage of NSPCs such as quiescence, proliferation, and differentiation (Meng et al., 2024). However, only few studies have examined metabolic regulators of neurogenesis (Knobloch et al., 2017; Petrelli et al., 2023; Stoll et al., 2015; Wani et al., 2022). Specifically, several metabolic features have been reported including fatty acid oxidation as a hallmark of quiescence in hippocampal NSPCs (Knobloch et al., 2017; Wani et al., 2022). Knobloch et al., using *in vitro* approaches, demonstrated that quiescent NSPCs express high levels of CPT1a and extensively oxidized palmitic acid (Knobloch et al., 2017). Inhibition of fatty acid oxidation using etomoxir as a CPT1a blocker resulted in extensive cell death of both quiescent and proliferative NSPCs (Knobloch et al., 2017). While indeed etomoxir inhibits CPT1a activity, it significantly impacts peroxisomal proteins, altering the ability of ω-oxidation, and affecting several mitochondrial proteins (Choi et al., 2024; Moon et al., 2024). Using Spot14-driven CreER mouse, Knobloch and colleagues attempted to elucidate the role of fatty acid oxidation on a specific population of NSPCs; however, it was not possible due to low recombination efficiency and sparse labeling (Knobloch et al., 2017). Previous studies have shown that CPT1a is expressed in NSPCs and co-localized with Nestin in the developing and adult brain (Jernberg et al., 2017; Knobloch et al., 2017). Since CPT1 is encoded by multiple genes (*Cpt1a*, *Cpt1b*, and *Cpt1c*) and CPT2 is encoded by a single gene and is universally expressed in all tissues, we generated a nestin-Cre CPT2 knockout mouse (CPT2^B−/−^) to examine the role of fatty acid oxidation on hippocampal NSPCs (Bonnefont et al., 2004; Jernberg et al., 2017).

In the present study, we observed that loss of CPT2 in the naive murine brain resulted in increased expression of several genes (*Ki67*, *Mash1*, *Dcx*, and *NeuroD*) involved with proliferation and subsequent neurogenesis in males. However, these changes in mRNA levels did not affect levels of expression of Ki67^+^ or ultimately number of hippocampal NeuN^+^ cells. These results are consistent with a previous report by Stoll and colleagues, who showed that inhibition of fatty acid oxidation did not affect proliferation in the hippocampal dentate gyrus by quantifying number of Ki67 positive cells (Stoll et al., 2015). It is well established that in addition to fatty acid oxidation, quiescent cells rely significantly on glycolysis (Wani et al., 2022). It is possible, that loss of CPT2 in NSPCs may increase expression of genes and proteins responsible for glycolysis in order to maintain quiescence, which should be evaluated in future studies.

Knobloch et al. showed that almost all CPT1A expressing cells are positive for SOX2, indicating that fatty acids are important, specifically in quiescence (Knobloch et al., 2017). Interestingly, in our study, analysis of female CPT2^B−/−^ brains showed that the number of YFP^+^SOX2^+^ was increased, while expression of YFP^+^Ki67^+^ cells and total number of NeuN^+^ cells were decreased in the dentate gyrus of the hippocampus without changes in mRNA expression. Behavioral assessment showed that female CPT2^B−/−^ mice display increased thigmotaxis in the open field and poor performance in the novel object recognition test compared to control female mice. No such deficits were observed in control and CPT2^B−/−^ male mice. These behavioral deficits are similar to previously reported and suggest that loss of CPT2 in NSPCs may be a subject of sex-differences (White et al., 2020).

Accumulating evidence suggests that sex is a biological variable contributing towards regulation of neurogenesis (Hillerer et al., 2013; Yagi et al., 2020). Yagi et al. reported that, under basal conditions, males and females have comparable number of Ki67^+^ and SOX2^+^ cells in the dentate gyrus of the hippocampus; however, they differed in subsequent stages of proliferation and maturation, with males showing faster maturation and greater attrition of newly generated neurons (Yagi et al., 2020). Our comparison of control and CPT2^B−/−^ mice did not show differences in the number of neurons, astrocytes, and oligodendrocytes in the dentate gyrus of adult males and were comparable to control females. Hillerer et. al reported that chronic stress leads to sex-specific alterations (Hillerer et al., 2013). Specifically, using a restrain stress model in Wistar rats, this group showed that males had decreased proliferation and increased quiescence, whereas in females these parameters were not affected by chronic stress, although the survival of newly generated cells was reduced (Hillerer et al., 2013). Whether mild TBI – traumatic and stressful condition – affects adult hippocampal neurogenesis and to which extent fatty acid oxidation may regulate this neurogenic response has not been delineated.

A large number of studies exploring neurogenesis in the TBI field have focused on moderate-to-severe TBI (Braun et al., 2002; Chirumamilla et al., 2002; Dash et al., 2001; Rola et al., 2006). Recently, Clark and colleagues examined a time course of neurogenesis in murine male dentate gyrus using lateral fluid percussion as a model of mTBI (Clark et al., 2020). They reported that mTBI, similar to severe TBI, induces proliferation that peaks on day 3, raising questions about the fate and significance of these newly generated cells (Clark et al., 2020). Using a different model of mTBI (closed head injury model), our results similarly show that in males, proliferation peaks on day 3 post-injury, as determined by the number of BrdU^+^, Ki67^+^ and BrdU^+^/Ki67^+^ cells. In contrast, hippocampal proliferation in females following mTBI was comparable to that in sham female mice – a response similar to the one observed following stress (Hillerer et al., 2013). Male CPT2^B−/−^ showed even further increase in proliferation compared to injured control mice, while in females no difference was detected between control and CPT2^B−/−^ injured brains. Thus, these findings once again underscore that there are sex-differences in proliferation following injury. Specifically, males upregulate proliferation in the dentate gyrus, which is further enhanced by loss of fatty acid oxidation, whereas female response remained blunted and was not modified by loss of fatty acid oxidation. Together these results show that both sex and metabolic changes impact proliferation after TBI.

Next, we assessed whether loss of fatty acid oxidation affects the differentiation and survival of these newly generated cells. Specifically, we examined brains at 28 days post-injury and quantified the number of BrdU^+^NeuN^+^ (neurons) and BrdU^+^GFAP^+^ (astrocytes) cells in the hippocampus. Our results show that there were no differences observed in the number of post-injury cells compared to sham controls and between genotypes. In addition, we quantified cells generated 24 hours before mTBI (pre-injury) and determined that the survival of these cells was not affected by trauma. Similar to the post-injury experiment, the number of BrdU^+^NeuN^+^ and BrdU^+^GFAP^+^ cells was comparable between control and CPT2^B−/−^ male and female mice. Together, in this model of brain trauma, we show that long-term survival of these newly generated cells was decreased, and we did not observe a shift towards astrogliosis. These findings are in agreement with previous studies showing that long-term survival of NSPCs post-injury is decreased, but contradict others (Dash et al., 2001; Downing et al., 2025). The opposing findings are in part due to different models of TBI, (i.e. closed head injury vs lateral fluid percussion injury vs controlled cortical impact), severity of injury, and age (Badner & Cummings, 2022; Clark et al., 2020; Ngwenya & Danzer, 2018; Wang et al., 2016). Another unresolved issue remains the differentiation of these newly generated cells, with studies demonstrating a shift towards astrogliosis while others reporting enhanced neurogenesis accompanied by abnormal dendritic formation (Bielefeld et al., 2024; Campbell et al., 2024; Clark et al., 2020; Neuberger et al., 2017). These diverse outcomes raise the question whether local metabolic milieu characteristics affect these outcomes (differentiation and long-term survival) post-trauma and should be explored in future studies.

In this study, we isolated one of the central metabolic pathways – fatty acid oxidation – and using a genetic approach, we have shown that proliferation can be enhanced while differentiation remains unaffected following TBI. Fatty acid oxidation is not limited to quiescent NSPCs but is also present in astrocytes and macrophages recruited into the brain post-injury (Jernberg et al., 2017; Morant-Ferrando et al., 2023; Nomura et al., 2016; White et al., 2020). While emerging evidence suggest that disrupted lipid metabolism is involved in inflammation, these factors can contribute to decreased survival of NSPCs. Future studies focused on delineating the mechanisms that promote endogenous repair are needed and should target pathways responsible for migration, differentiation, and proper integration. These interventions may include - but are not limited to - augmenting levels of neurotrophic factors, implementing supplement and nutraceutical therapies, and applying non-invasive brain stimulation techniques with consideration of sex differences and timing of administration (Aguilar-Arredondo & Zepeda, 2025; Conti et al., 2024; Lucke-Wold et al., 2025; O’Leary et al., 2025; Sun, 2014).

In summary, in this study, loss of brain fatty acid oxidation in Nestin^+^ neural stem and progenitor cells did not affect adult hippocampal proliferation and neurogenesis under basal conditions. Abnormal hippocampal neurogenesis has been linked to cognitive deficits after mild traumatic brain injury (Ngwenya & Danzer, 2018). Thus, a better understanding and enhancing endogenous hippocampal neurogenesis could improve neurological outcomes. Using close head injury as a model of mTBI, we demonstrated that hippocampal neurogenesis is upregulated, and that the loss of fatty acid oxidation in Nestin^+^ NSPCs further enhances the endogenous proliferative response post-injury in males only. These findings highlight the importance of investigating sex-specific metabolic features in post-traumatic hippocampal neurogenesis. Understanding these important factors may promote specific targeted approaches towards improving cognitive function after TBI.

## Supplementary Material

Supplemental Material

Supplemental Material

Supplemental Material
