# Supplementary figures and images for: Loss of Fatty Acid Oxidation by Neural Stem and Progenitor Cells Increases Proliferation but Does Not Improve Long-Term Neurogenesis After Mild Traumatic Brain Injury
Source: ASN Neuro. 2026 Jan 18;18(1):2610198. doi: 10.1080/17590914.2025.2610198 (PMC12818800; doi:10.1080/17590914.2025.2610198)

**A**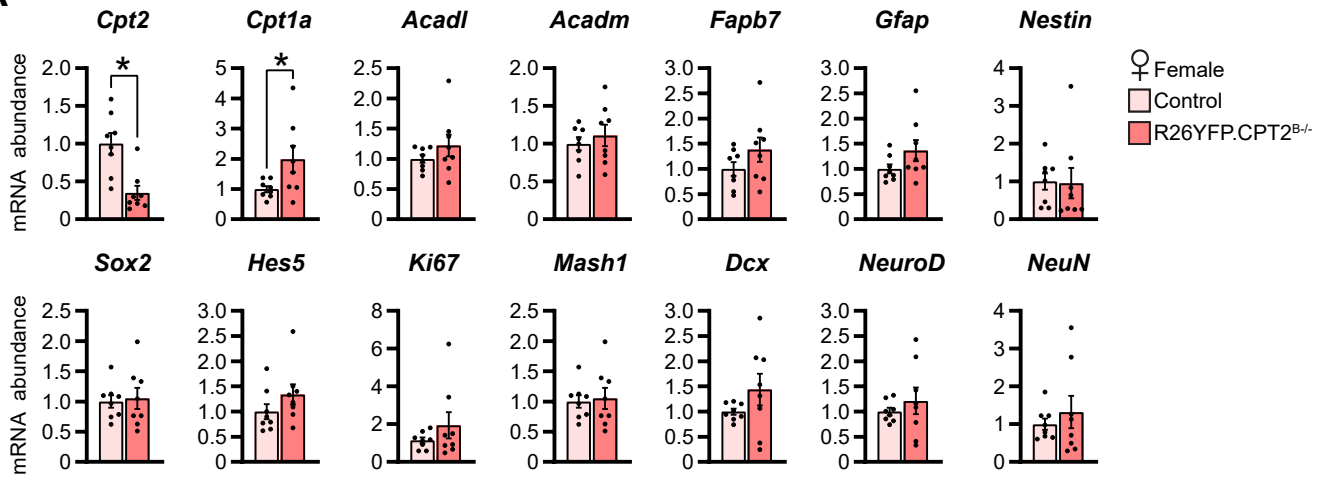**B**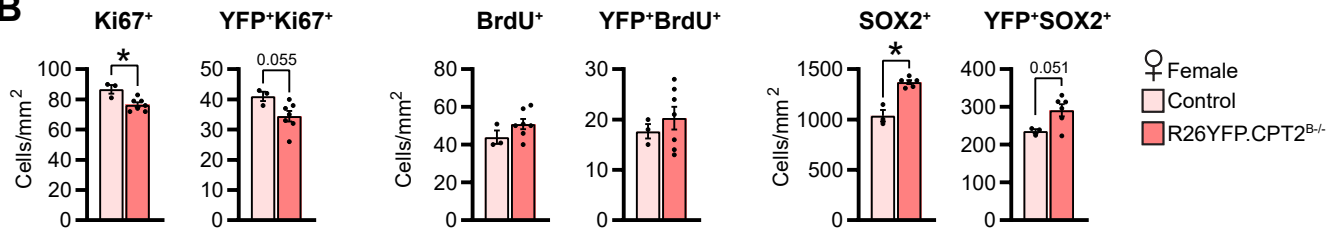

Supplement: Supplemental Material [file TASN_A_2610198_SM4211.pdf]

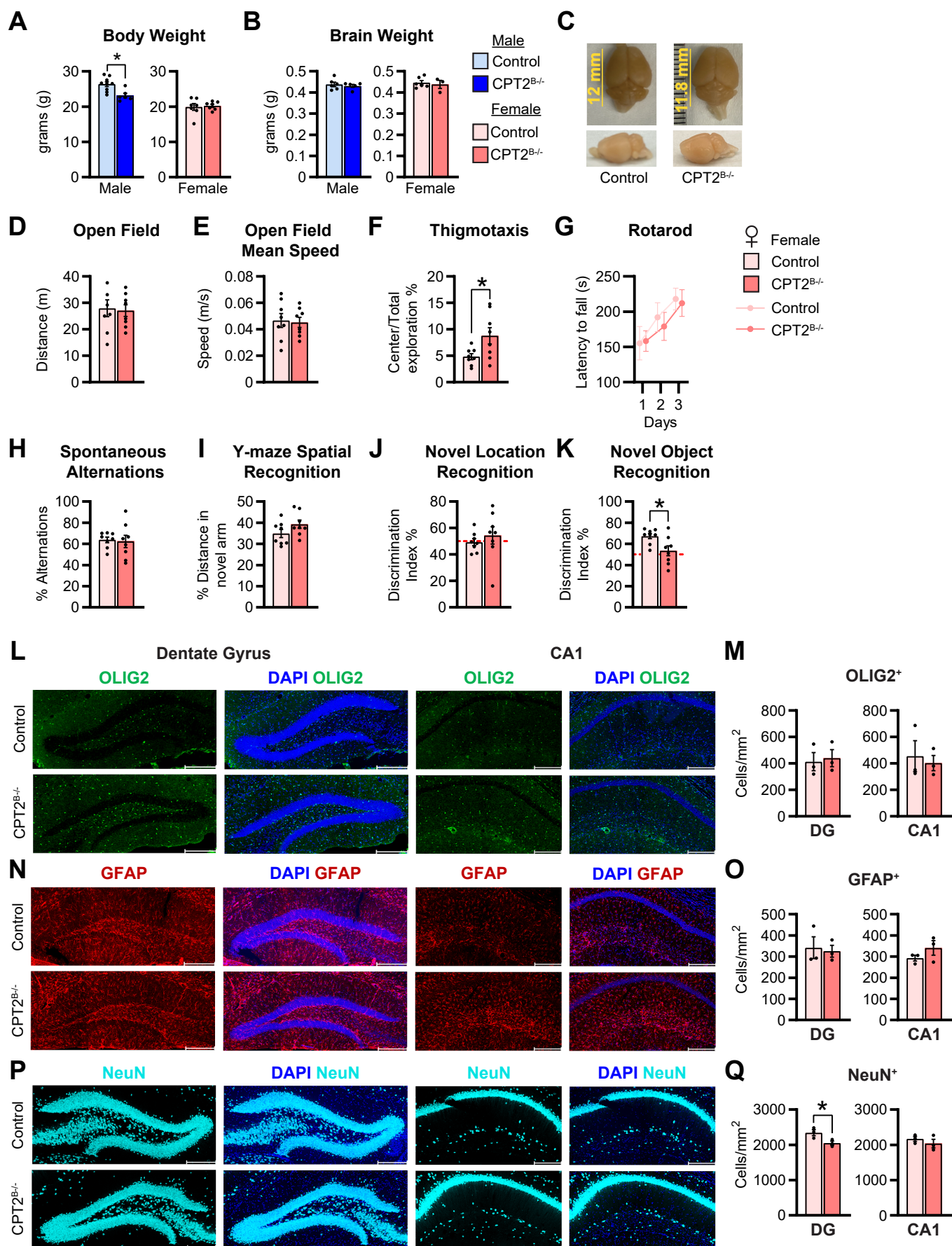

Supplement: Supplemental Material [file TASN_A_2610198_SM3752.pdf]
