# Supplementary material for: Loss of Fatty Acid Oxidation by Neural Stem and Progenitor Cells Increases Proliferation but Does Not Improve Long-Term Neurogenesis After Mild Traumatic Brain Injury
Source: ASN Neuro. 2026 Jan 18;18(1):2610198. doi: 10.1080/17590914.2025.2610198 (PMC12818800; doi:10.1080/17590914.2025.2610198)

A

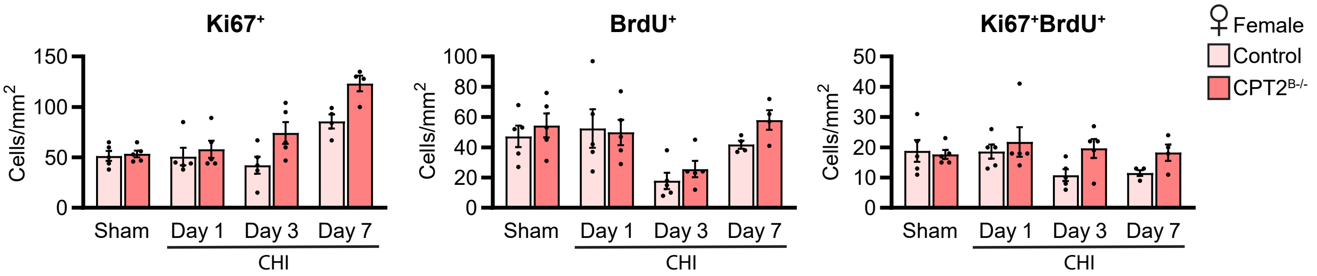

B

	Comparisons	Adj. *p*-value		
		Ki67⁺	BrdU⁺	Ki67⁺BrdU⁺
Control	Sham vs. CHI 1d	>0.9999	>0.9999	>0.9999
	Sham vs. CHI 3d	>0.9999	0.0615	0.362
	Sham vs. CHI 7d	**0.0338***	>0.9999	0.6209
	CHI 1d vs. CHI 3d	>0.9999	**0.0182***	0.3999
	CHI 1d vs. CHI 7d	**0.0297***	>0.9999	0.6771
	CHI 3d vs. CHI 7d	**0.0042***	0.263	>0.9999
$CPT2^{B-/-}$	Sham vs. CHI 1d	>0.9999	>0.9999	>0.9999
	Sham vs. CHI 3d	0.3901	0.0703	>0.9999
	Sham vs. CHI 7d	**<0.0001***	>0.9999	>0.9999
	CHI 1d vs. CHI 3d	0.8771	0.1894	>0.9999
	CHI 1d vs. CHI 7d	**<0.0001***	>0.9999	>0.9999
	CHI 3d vs. CHI 7d	**0.0011***	**0.0473***	>0.9999

C

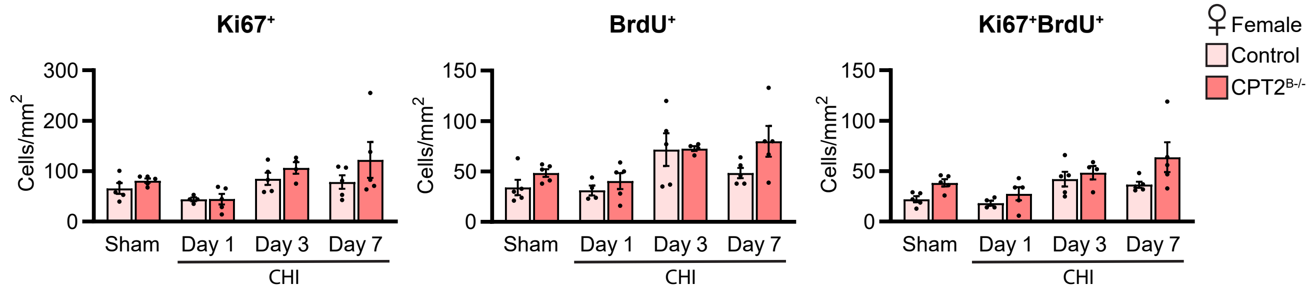

D

	Comparisons	Adj. *p*-value		
		Ki67⁺	BrdU⁺	Ki67⁺BrdU⁺
Control	Sham vs. CHI 1d	>0.9999	>0.9999	>0.9999
	Sham vs. CHI 3d	>0.9999	0.0524	0.3238
	Sham vs. CHI 7d	>0.9999	>0.9999	0.9544
	CHI 1d vs. CHI 3d	0.5849	0.0473	0.1882
	CHI 1d vs. CHI 7d	0.9556	>0.9999	0.5649
	CHI 3d vs. CHI 7d	>0.9999	0.5461	>0.9999
$CPT2^{B-/-}$	Sham vs. CHI 1d	0.6989	>0.9999	>0.9999
	Sham vs. CHI 3d	>0.9999	0.5945	>0.9999
	Sham vs. CHI 7d	0.4813	0.161	0.0973
	CHI 1d vs. CHI 3d	0.0851	0.1797	0.3422
	CHI 1d vs. CHI 7d	**0.0107***	**0.0376***	**0.0063***
	CHI 3d vs. CHI 7d	>0.9999	>0.9999	0.9547

E

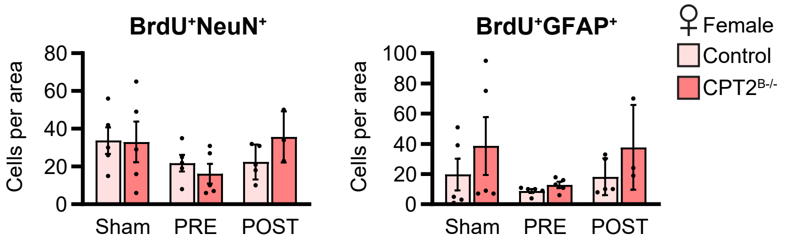

Supplement: Supplemental Material [file TASN_A_2610198_SM3751.pdf]
